# Complement Receptor 3-Mediated Inhibition of Inflammasome Priming by Ras GTPase-Activating Protein During *Francisella tularensis* Phagocytosis by Human Mononuclear Phagocytes

**DOI:** 10.3389/fimmu.2018.00561

**Published:** 2018-03-26

**Authors:** Ky V. Hoang, Murugesan V. S. Rajaram, Heather Marie Curry, Mikhail A. Gavrilin, Mark D. Wewers, Larry S. Schlesinger

**Affiliations:** ^1^Center for Microbial Interface Biology, Department of Microbial Infection and Immunity, The Ohio State University, Columbus, OH, United States; ^2^Division of Pulmonary, Allergy, Critical Care and Sleep Medicine, Department of Internal Medicine, Davis Heart & Lung Research Institute, The Ohio State University, Columbus, OH, United States; ^3^Texas Biomedical Research Institute, San Antonio, TX, United States

**Keywords:** *Francisella*, immune suppression, Ras GTPase-activating protein, inflammasome, complement receptor, caspase-1

## Abstract

*Francisella tularensis* is a remarkably infectious facultative intracellular bacterium of macrophages that causes tularemia. Early evasion of host immune responses contributes to the success of *F. tularensis* as a pathogen. *F. tularensis* entry into human monocytes and macrophages is mediated by the major phagocytic receptor, complement receptor 3 (CR3, CD11b/CD18). We recently determined that despite a significant increase in macrophage uptake following C3 opsonization of the virulent Type A *F. tularensis* spp. *tularensis* Schu S4, this phagocytic pathway results in limited pro-inflammatory cytokine production. Notably, MAP kinase/ERK activation is suppressed immediately during C3-opsonized Schu S4-CR3 phagocytosis. A mathematical model of CR3-TLR2 crosstalk predicted early involvement of Ras GTPase-activating protein (RasGAP) in immune suppression by CR3. Here, we link CR3-mediated uptake of opsonized Schu S4 by human monocytes and macrophages with inhibition of early signal 1 inflammasome activation, evidenced by limited caspase-1 cleavage and IL-18 release. This inhibition is due to increased RasGAP activity, leading to a reduction in the Ras-ERK signaling cascade upstream of the early inflammasome activation event. Thus, our data uncover a novel signaling pathway mediated by CR3 following engagement of opsonized virulent *F. tularensis* to limit inflammasome activation in human phagocytic cells, thereby contributing to evasion of the host innate immune system.

## Introduction

*Francisella tularensis* is a facultative intracellular bacterium that causes tularemia, a zoonotic disease that is transmitted through aerosol or arthropod vectors (1–4). The pneumonic form of the highly virulent Type A *F. tularensis* Schu S4 strain can lead to a fatal disease if left untreated even with an inoculum of less than 10 colony-forming units (1). *Francisella* infects several cell types; however, the primary target of *F. tularensis* is the macrophage, particularly the alveolar macrophage during airborne infection (5). Compared to the nonpathogenic subspecies [e.g., *Francisella novicida* (*Fn*)], the Schu S4 strain leads to significantly reduced pro-inflammatory cytokine production (6–9). This reduced response during the early stage of infection is critical for establishment of infection in human macrophages.

The host has evolved multiple strategies to recognize pathogen-associated molecular patterns (PAMPs) by membrane-bound and cytosolic germline-encoded pattern recognition receptors (PRRs) (10–12). PRR engagement leads to the expression and production of various anti-microbial components and cytokines through the nuclear factor κB (NF-κB) signaling pathway (13). Cytoplasmic PRRs consist of the nucleotide-binding oligomerization domain receptors (NOD)-like receptors (NLRs), TLR-7, 8, and 9, retinoic-acid inducible gene-I-like helicase, AIM2, and the PYRIN protein families. Activation of NLRs, AIM2, and the PYRIN protein families initiates assembly of a cytoplasmic complex known as the inflammasome that serves as an activation platform for the mammalian cysteine protease caspase-1 (10). Caspase-1 activation induces the proteolytic cleavage of pro-inflammatory cytokines IL-1β and IL-18 (13). As a member of the NOD-like receptor family, the most widely studied NLRP3 inflammasome responds to a variety of structurally and chemically diverse molecules (12, 13), and TLR signaling and inflammasome activation often crosstalk during microbial infection (14–17). Activation of the NLRP3 inflammasome in monocytes, macrophages, dendritic cells, and microglial cells requires two steps: the first priming step is mediated by PAMPs-PRRs and the second step mediated by various bacterial toxins or ATP, and potassium efflux, leads to inflammasome assembly and activation (18–21). Importantly, *de novo* synthesis of pro-IL-18 and upregulation of NLRP3 are not necessary for NLRP3 inflammasome activation in response to TLR-induced priming by LPS (17, 22, 23). Recent studies demonstrate that NLRP3 inflammasome priming by LPS is dependent on MAP kinase (MAPK)/ERK activation (24) and proteasome function (16, 24).

Macrophages combat *F. tularensis* infection primarily by generating TLR2-dependent pro-inflammatory cytokines [e.g., TNF, IL-1β, Ref. (14, 25–27)] and inflammasome activation (5, 14, 26, 28–31). Inflammasome activation by *Francisella* requires two distinct signals: a priming signal and an NLR-dependent sensing step. In mice, the NLR component is mainly due to the activation of AIM2 (14, 32, 33). However, in human monocytes and monocyte-derived macrophages (MDM) the key NLRs appear to be NLRP3 and pyrin (34–39). Nevertheless, regardless of the NLR component involved, there is mounting evidence that inflammasome priming is an independent required event in caspase-1 activation that happens rapidly, triggered by PAMPs, within min of pathogen contact and does not require new gene transcription (16, 17, 22, 23). This priming step is dependent in part on ERK signaling and proteasome function (16). In human monocytes infected by *Francisella*, inflammasome priming is TLR2 and ERK signaling dependent (40).

Complement receptor 3 (CR3; CD11b/CD18) belongs to the β_2_-integrin family primarily expressed in phagocytic cells. It provides a highly effective and safe mode of entry for many pathogens (41–43) by enabling subversion of host immune responses and evasion of intracellular killing (42, 44–48). Complement factor C3 opsonization of Schu S4 is critical for uptake by human macrophages in a CR3-dependent manner, which leads to immunosuppression and increased bacterial survival (49–51). CR3’s function is dependent on activation of outside-in and inside-out two-way signals (52). Signaling crosstalk between CRs and TLRs occurs (53). Our previous study (54) showed that outside-in signaling through engagement of CR3 by C3-opsonized Schu S4 inhibits the TLR2 signaling cascade, resulting in a relatively silent mode of entry. CR3-mediated signaling activates Lyn kinase and AKT which leads to an increased expression of MKP-1, downregulating MAPK activity over 30 min. This allows for increased phagocytosis simultaneously with a dampened host immune response (54). By contrast, in the absence of serum, PAMPs (e.g., lipoproteins) are primarily recognized by TLR2 which activates MAPK (ERK1/2 and p38) and NF-κB signaling pathways, leading to robust pro-inflammatory responses despite limited phagocytosis (54). Although Lyn kinase and MKP-1 are essential components of the CR3 pathway that limits TLR2 activity, additional upstream signaling components that act upon receptor engagement remain unknown and may be linked to inflammasome priming.

A mathematical model of CR3/TLR2 crosstalk in the context of *F. tularensis* infection (55) predicted that phagocytosis-associated changes in cell membrane composition, primarily by CR3, can inhibit ERK activation *via* p120 Ras GTPase-activating protein (RasGAP). More specifically, non-opsonized Schu S4 primarily activates TLR2 which leads to the sequential activation of RasGTPase, Raf, and ERK. By contrast, ligation of C3-opsonized Schu S4 by CR3 results in the activation of Lyn kinase which the model predicts recruits RasGAP, a negative regulator Ras. Thus, we experimentally sought to test whether inhibition of ERK by C3-opsonized Schu S4 is mediated by increased RasGAP activity. Herein, we show that opsonized Schu S4 uptake by CR3 inhibits step 1 inflammasome priming evidenced by limited caspase activity and IL-18 release. This inhibition is, in part, due to increased RasGAP activity, which leads to a reduction in Ras-ERK activation. Thus, our data uncover a novel signaling pathway initiated by CR3 following engagement of opsonized virulent Schu S4 that results in evasion of the host innate immune system. Together with prior work, CR3-mediated phagocytosis of *Francisella* represents an important bacterial survival mechanism activating multiple checkpoints to limit early pro-inflammatory responses.

## Materials and Methods

### Bacterial Strains and Growth Conditions

The *F. tularensis* subspecies *tularensis* Schu S4 strain (*Ft*) was a generous gift from Dr. Rick Lyons at Colorado State University and *F. novicida* U112 (*Fn*) was purchased from ATCC. Bacterial strains were cultured on chocolate agar II pates (Becton Dickinson, Sparks, MD, USA) for 48 h (*Ft*) or 24 h (*Fn*) at 37°C (54).

### Human Monocyte and Monocyte-Derived Macrophage (hMDM) Preparation

Human peripheral blood was obtained from healthy donors (with no known exposure to *Francisella*) *via* venipuncture following a protocol approved by the Ohio State University Institutional Review Board. Peripheral mononuclear cells (PBMCs) were isolated from heparinized blood as previously described (54) over a Ficoll cushion (GE Healthcare Bio-Science, Piscataway, NJ, USA). The PBMCs were then washed with RPMI 1640 plus l-glutamine (Gibco-Life Technologies, Grand Island, NY, USA). Monocytes were separated from PBMCs using positive selection with anti-CD14 coated magnetic beads, following the manufacturer’s instructions (Miltenyi Biotiec San Diego, CA, USA) and were maintained in RHH [RPMI 1640 with l-glutamine, 10 mM HEPES, and 0.25% human serum albumin (HSA)] as described (56). For hMDMs, PBMCs were cultured in Teflon wells with RPMI 1640 plus l-glutamine containing 20% autologous serum at 37°C for 5 days (54). Cells were recovered from Teflon wells and plated in the presence of 10% autologous serum in 6-well or 24-well tissue culture plates for 2–3 h at 37°C. Lymphocytes were then washed away leaving hMDM monolayers (~2.0 × 10^5^ cells/well for 24-well plates and ~1 × 10^6^ cells/well for 6-well plates, 99% pure) for *Francisella* infection. Autologous sera were isolated from healthy human blood with no known exposure to *Francisella via* venipuncture. Sera were prepared and stored as previously described (57) to preserve complement activity.

### Reagents and Antibodies (Abs)

Antibodies against phospho-ERK1/2 and total ERK1/2 were purchased from Cell Signaling (Boston, MA, USA). Caspase-1 Ab (anti-human rabbit polyclonal antisera) was described in a previous study (16). Abs against β-actin and RasGAP were from Santa Cruz Biotechnology (Santa Cruz, CA, USA). Phospho-Dok-1 (pTyr362) was from Acris Antibodies, Inc. (San Diego, CA, USA). Ab specific to phospho-Lyn (Y396) and mouse mAb against *F. tularensis* LPS (FB11) were from Abcam (Cambridge, MA, USA). Rat monoclonal Ab (M1/70) and mouse mAb (H5A4) against CD11b were from the Developmental Studies Hybridoma Bank (University of Iowa, Iowa City, IA, USA). ATP was purchased from Sigma-Aldrich (St. Louis, MO, USA). Secondary Abs conjugated with AF-488, AF-549 (anti-rabbit), and AF-647 (anti-mouse) were from Life Technology (Invitrogen, Carlsbad, CA, USA). Human C3-depleted serum and human purified C3 were purchased from Comptech (Tyler, TX, USA). C3 was repleted at 1 mg/ml in C3-depleted serum. Control scrambled small interfering RNA (siRNA) and pre-siGenome smartpool targeting CD11b or RasGAP were obtained from Thermo Scientific Dharmacon RNA Technologies (Lafayette, CO, USA).

### Monocyte siRNA Transfection

Peripheral mononuclear cells were transfected with scrambled siRNA or gene-specific siRNAs by electroporation using the human Monocyte Nucleofector Kit (VPA-1007, Lonza) following the manufacturer’s protocol with the following modifications. Briefly, 1.0 × 10^7^ PBMCs were transfected with 200 nM of the targeting siRNA (CD11b or RasGAP) or a non-targeting scrambled siRNA. Transfected PBMCs were re-suspended in 2 ml of RPMI plus Glutamax supplemented with 20% autologous serum and monocytes were plated onto tissue culture plates for 3–4 h at 37°C in 5% CO_2_. Lymphocytes were removed by washing three times, leaving the monocyte monolayer at ~1.0 × 10^6^ cells/well for *Francisella* infection. Gene knockdown was confirmed by Western blot for target proteins.

### Bacterial Opsonization, Monocyte and hMDM Infection, and Cell Lysis

*Schu S4 and Fn* were harvested, washed with PBS and adjusted to an OD at 600 nm (OD_600_) of 0.4, equivalent to 3 × 10^9^ CFU/ml. Bacteria were then pre-opsonized with 10% autologous serum, heat inactivated (HI) autologous serum, C3-depleted serum, or C3-repleted serum in RHH (RPMI 1640 with l-glutamine, 10 mM HEPES, and 0.25% HSA) at 37°C while shaking (54). After 30 min incubation, the opsonized bacteria were washed three times and re-suspended in RHH to the desired bacterial concentrations. Non-opsonized bacteria were incubated with RHH instead of serum and prepared side by side with pre-opsonized bacteria.

*Francisella* infection of hMDM monolayers was performed as described (54). Briefly, day 5 hMDM monolayers were washed extensively and non-opsonized or pre-opsonized bacteria were added to the hMDM monolayers at an MOI of 100 in 1 ml RHH. The infection was synchronized by centrifugation at 250 × *g* for 10 min at 4°C followed by incubation at 37°C in 5% CO_2_. At the indicated time points, cells were washed with ice cold PBS and lysed with TN-1 lysis buffer [50 mM Tris (pH 8.0), 10 mM EDTA, 10 mM Na4P_2_O_7_, 10 mM NaF, 1% Triton X-100, 125 mM NaCl, 10 mM Na_3_VO_4_, and 10 µg/ml each of aprotinin and leupeptin] while on ice for 5 min (54). The lysates were then passed through a 27 G needle and filtered using a 0.22 µm cellulose acetate membrane (Costa, Corning, NY, USA) by centrifugation at 16,000 × *g* at 4°C for 5 min. Protein concentrations of the cell lysates were measured using the Pierce BCA-protein assay kit (Thermo Scientific, Rockford, IL, USA). Samples were used for examination of active RasGAP or subjected to separation by SDS-PAGE and analyzed by Western blot for the proteins of interest.

### Assay of Inflammasome Priming

Examination of inflammasome priming by *Francisella* was performed with monocytes either in suspension (1.5 ml microcentrifuge tube) or adhered to a 6-well tissue culture plate (~10^6^cells/treatment). Non-opsonized or pre-opsonized *Francisella* (MOI of 100) was added in 1 ml RHH, and infection was synchronized by centrifugation at 250 × *g* for 10 min at 4°C (for monolayer experiments) followed by incubation at 37°C. After 30 min, ATP was added (5 mM) for 30 min. The supernatants were collected in 1.5 ml microfuge tubes and centrifuged for 5 min at 4°C at 250 × *g* to remove floating cells. Supernatants were examined for IL-18 by ELISA and active caspase-1 by Western blot.

### Western Blotting and IL-18 ELISA

Protein matched cell lysates were resolved by SDS-PAGE and transferred onto a nitrocellulose membrane (BioRad, CA, USA), probed with the Abs of interest, followed by HRP-conjugated second Ab and developed by ECL (GE Healthcare, NY, USA) using autoradiography.

Mature IL-18 in the supernatants was quantified by sandwich ELISA using MBL Abs as described (58) with the following modifications. Anti-human IL-18 (MBL International, mouse IgG2A mAb) was coated onto a 96-well Costar plate (Fischer Scientific Inc., Rockford, IL, USA) overnight at 4°C. Plates were washed three times with PBS + 0.5% Tween 20, blocked with 5% BSA in PBS for 1 h at 37°C, and then incubated with samples or recombinant IL-18 standard (MBL International). Biotin-labeled anti-human IL-18, 1:1,000 (MBL International, rat IgG2A mAb) was then added for 2 h at 37°C, washed four times with PBS + 0.5% Tween 20 and Streptavidin-HRP (eBiosciences) added for 1 h. The plate was developed using TMB Peroxidase Substrate and Peroxidase Substrate Solution B (BD Sciences, Sparks, MD, USA). After 10 min, 0.2 N H_2_SO_4_ was added to stop the reaction. Plates were read on a Perkin Elmer 2030 Victor X3 Multilabel Reader, measuring absorbance at 450 nm after subtracting background 630 nm absorbance. This ELISA is highly specific for mature IL-18 (16).

### Assay of RasGAP Activity

Activation status of the guanosine triphosphate (GTP)-binding protein Ras is dictated by the relative intensities of two opposing reactions: (1) formation of active Ras–GTP complexes, stimulated by guanine-nucleotide exchange factors (GEFs) and (2) Ras conversion to the inactive form Ras–GDP as a result of the deactivating action of GTPase-activating proteins (GAPs) (59). We assayed for RasGAP activity indirectly by determination of Ras–GTP levels using an activated Ras interaction assay as described (60) following the manufacture’s protocol (Thermo Fisher Scientific Inc., Rockford, IL, USA). Briefly, 80 µg of GST-Raf1-RBD protein was mixed with 500 µg of cell lysate protein in the presence of glutathione resin agarose beads. The reaction mixture was incubated at 4°C for 1 h with gentle rocking. After removing unbound proteins by washing with buffer, the samples were collected by adding reducing sample buffer. The collected proteins were subjected to SDS-PAGE and Western blotting with anti-Ras Ab followed by HRP-conjugated second Ab and developed by ECL (BioRad, CA, USA) using autoradiography. Densitometry was evaluated using ImageJ software.

### Immunofluorescence Microscopy

Monocyte or hMDM monolayers were prepared on coverslips in a 24 well tissue culture plate. Phagocytosis of non-opsonized or pre-opsonized *Francisella* (MOI of 50) was synchronized at 4°C followed by incubation for 5 min at 37°C. Monolayers were washed to remove unbound bacteria and fixed with 4% paraformaldehyde followed by staining with Abs of interest (54, 61). In brief, cells were permeabilized with ice cold methanol for 10 s, blocked with 5% goat serum and 0.5% BSA in PBS for 2 h at room temperature (RT), incubated with the primary Abs (anti-CD11b, -RasGAP, -*Ft*, or -Dok-1 Abs) for 1 h at RT, washed, and then incubated with the appropriate fluorescence secondary Abs for 1 h at RT. Control experiments verified that the Abs do not bind directly to bacteria. Host nucleic acid was stained with 4′,6′-diamidino-2-phenylindole at RT for 5 min. Coverslips were mounted on glass slides and viewed on an Olympus FluoView 1000 confocal microscope.

### Statistical Analysis

Experiments were repeated at least three times with three different donors. Absolute results varied among donors but the patterns were the same relative to internal controls in each experiment. Statistical analysis was performed using GraphPad Prism 5. Data are presented as mean ± SD and *p*-values were calculated using one-way ANOVA for multiple comparisons and adjusted with Bonferroni’s correction. A *p*-value <0.05 was considered significant (*p < 0.05; **p < 0.001; ***p < 0.0001; NS, not significant).

## Results

### Serum Opsonization Mediates Inhibition of Inflammasome Priming by *Francisella* in Human Monocytes

Inflammasome activation involves a required priming step upon recognition of an invading microbe followed by inflammasome protein assembly resulting in caspase-1 activation and cleavage of pro-IL-1β and pro-IL-18 to generate biologically active forms of IL-1β and IL-18, respectively (12). Priming can be induced by TLR agonists (e.g., LPS) but full inflammasome activation requires a second signal such as a short burst of high dose ATP. Under these conditions caspase-1 activation leads to maturation of IL-18, which occurs independently of new protein synthesis (16, 40, 62). Importantly, *de novo* synthesis of pro-IL-18 is not necessary for NLRP3 inflammasome activation in response to TLR-induced priming (LPS) (17, 22, 23). Thus, IL-18 processing when studied early after infection can serve as a marker for inflammasome priming, defined as signaling events independent of new protein synthesis (16). In this context, inflammasome priming is dependent on ERK signaling (16), and we have previously found that serum opsonization of Schu S4 downregulates ERK activation within 5 min of *Francisella* phagocytosis (54).

**Figure 1:**
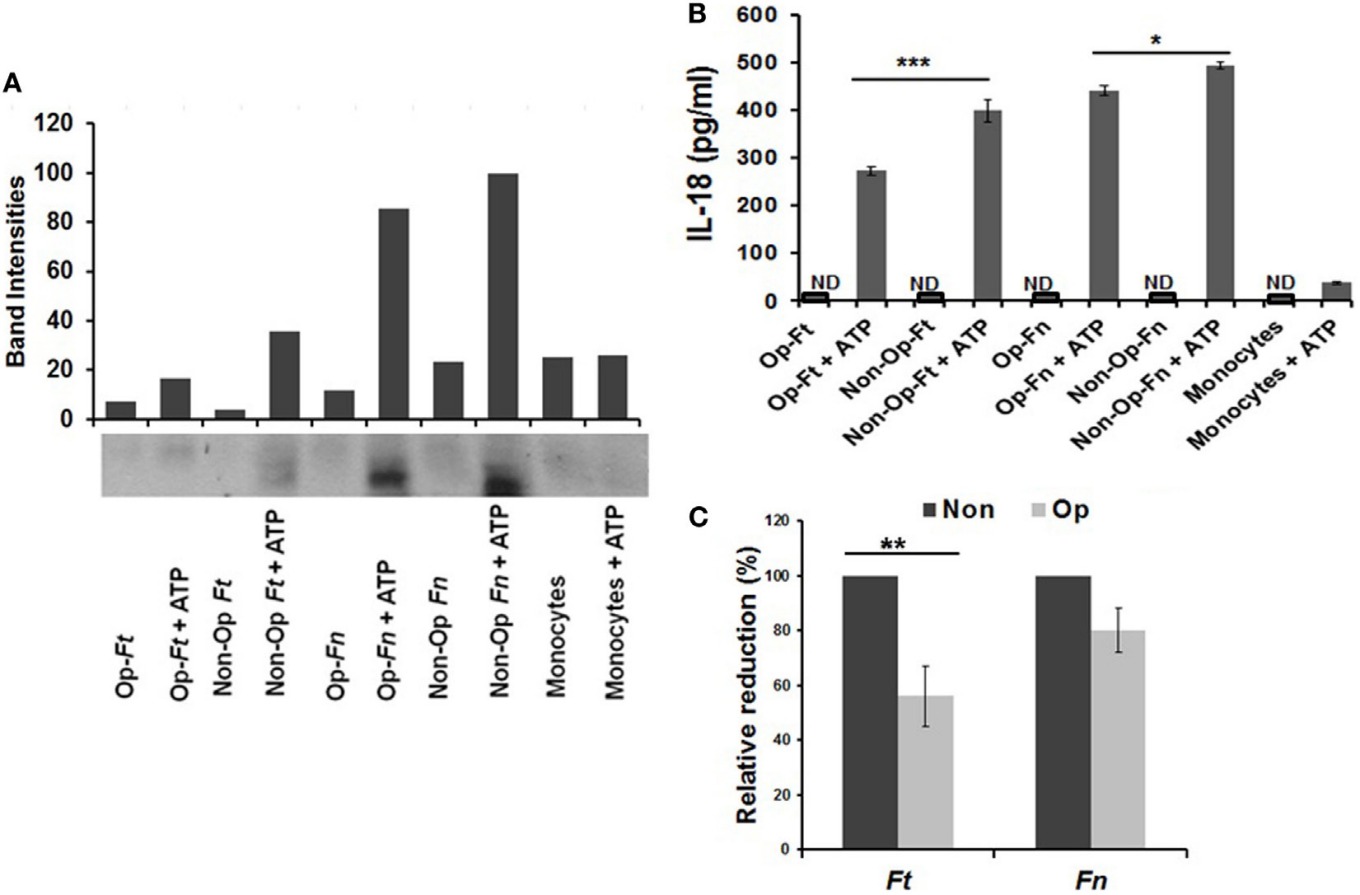
Serum opsonization of Schu S4 results in inhibition of inflammasome priming by human monocytes. Human monocytes (1 × 10^6^) were infected with either non-opsonized (Non) or autologous serum pre-opsonized (Op) Schu S4 or *Francisella novicida* (*Fn*) (MOI = 100). After 30 min incubation, ATP was added (5 mM) followed by incubation for an additional 30 min. The supernatants were collected and activated caspase-1 (p20) was examined by Western blot and densitometry using anti- caspase-1 antibody **(A)**. Quantification of IL-18 release was measured by ELISA **(B)**. Data are the representative of three independent experiments (mean ± SD). Cumulative data from three experiments for IL-18 reduction from pre-opsonized bacterial infection is presented as percentage relative to non-opsonized bacterial infection **(C)**. *p*-Values were calculated using one-way ANOVA for multiple comparisons. *p < 0.05; **p < 0.01; ***p < 0.001; ND, not detected.

Monocytes and hMDMs both highly express CR3 (54, 63). We first verified our previous finding with hMDMs (54) by demonstrating that serum pre-opsonized *Francisella* limits ERK activation in human monocytes at early time points and this limitation is significantly stronger for Schu S4 than *Fn* (Figure S1 in Supplementary Material). Next, we determined whether serum pre-opsonized *Francisella* (Schu S4 and *Fn*) inhibits inflammasome priming in human monocytes by examining caspase-1 activity and mature IL-18 release in the culture supernatants. As shown in Figure 1A (and Figure S2 in Supplementary Material), Schu S4 infection leads to minimal caspase-1 activation following ATP addition, whereas *Fn* infection leads to robust caspase-1 activation. Both types of pre-opsonized bacteria induce less IL-18 release than non-opsonized bacteria, although the finding is more pronounced for the virulent Schu S4 strain (Figures 1B,C). Together, these data provide evidence that serum opsonization limits inflammasome priming during *Francisella* infection of human monocytes.

### Complement C3 Opsonization of *Francisella* Mediates Limited Inflammasome Priming During Infection of Human Monocytes

**Figure 2:**
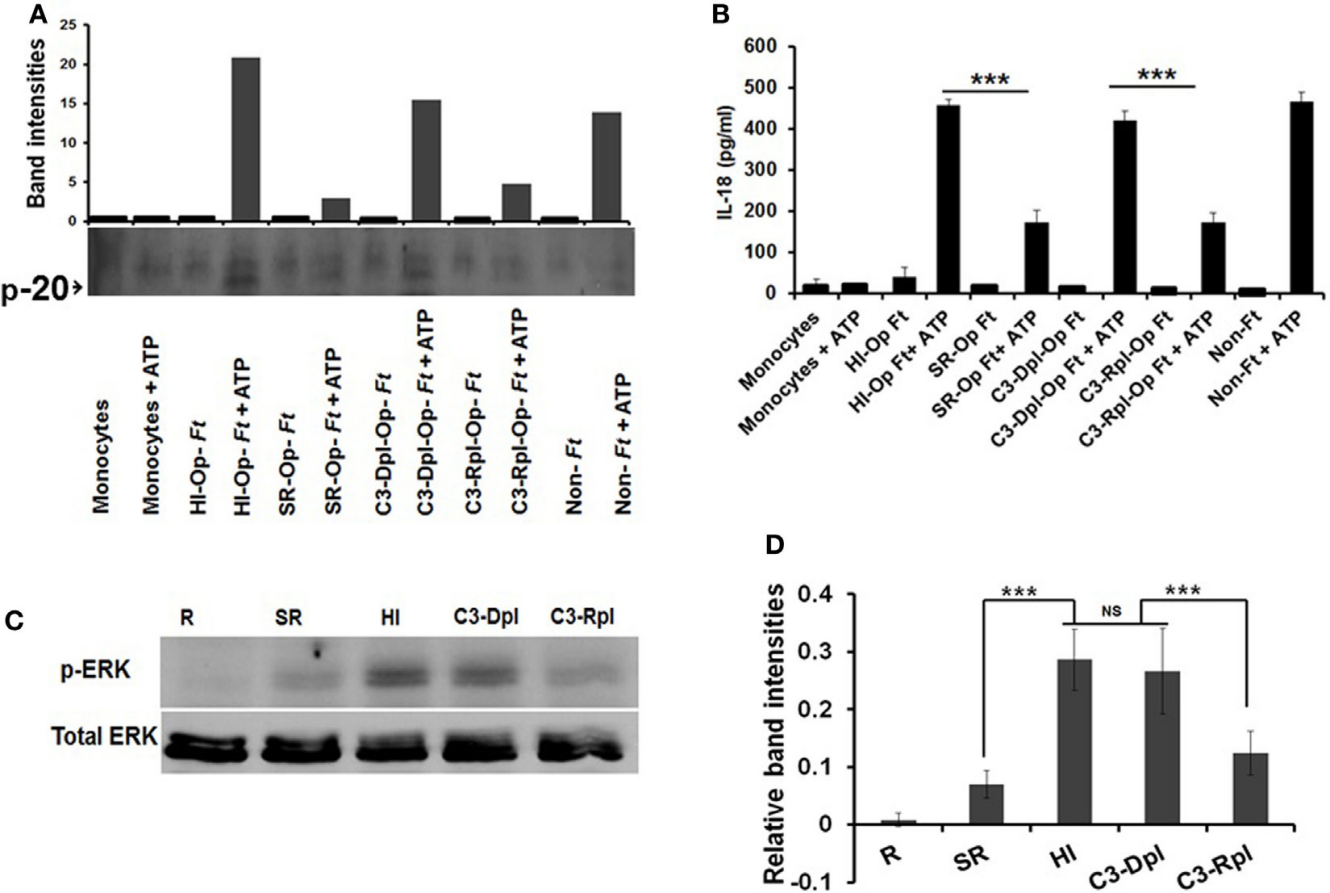
Complement protein 3 is required for Schu S4-mediated inhibition of inflammasome priming. Human monocytes (1 × 10^6^) were infected with Schu S4 that had been pre-opsonized in fresh autologous serum (SR), heat inactivated (HI) serum, complement C3-depleted serum (C3-Dpl), complement C3-repleted serum (C3-Rpl), or non-opsonized (MOI = 100) at 37°C. After 30 min, ATP was added (5 mM) for 30 min. Supernatants were collected and then subjected to caspase-1 Western blotting and densitometry **(A)** for p20 and IL-18 release by ELISA **(B)**. Cell lysates were subjected to Western blotting using anti-phospho-ERK Ab **(C)** and densitometry **(D)**. Data are representative of three independent experiments performed in duplicate (mean ± SD). ***p < 0.001; NS, not significant.

Complement C3 in serum is a major opsonin for *Francisella* (50) and can reduce bacterial-induced pro-inflammatory responses (54). Since serum opsonization limits inflammasome priming, we determined the specific role of C3 in this process. We infected human monocytes with Schu S4 that had been pre-opsonized in fresh autologous serum (SR), heat inactivated (HI) serum, complement C3-depleted serum (C3-Dpl), or complement C3-repleted serum (C3-Rpl), and measured caspase-1 activation and IL-18 release by Western blot and ELISA, respectively. As shown in Figures 2A,B (Figure S3 in Supplementary Material), caspase-1 activation was reduced during incubation with SR bacteria; by contrast, incubation with HI bacteria (heat inactivation abolishes complement activity) or C3-Dpl bacteria led to increased caspase-1 cleavage. Importantly, incubation with C3-Rpl bacteria restored the limited caspase-1 activation, which demonstrates the importance of C3 opsonization in this process. Similar to the caspase-1 activation results, incubation with SR or C3-Rpl Schu S4 led to limited IL-18 release whereas incubation with HI bacteria, C3-Dpl bacteria, or non-opsonized bacteria led to robust IL-18 release (Figure 2B).

Since activation of MAPK/ERK has been linked to inflammasome priming (16) and serum opsonization of Schu S4 downregulates ERK activation on hMDMs (54), we determined the level of ERK activation in monocyte cell lysates that were left uninfected or infected with Schu S4 that had been pre-opsonized with fresh serum (SR), HI serum, C3-Dpl serum, or C3-Rpl serum. Our results show that ERK activation is significantly reduced in fresh serum (SR) and C3-Rpl serum, confirming the important role of C3 opsonization in limiting ERK activation in human monocytes (Figures 2C,D). Taken together, our data demonstrate that inflammasome priming, indicated by early caspase-1 activation and IL-18 release during virulent Schu S4 infection is tightly controlled by complement C3 opsonization.

### CR3/CD11b Engagement Limits Inflammasome Priming During Schu S4 Infection

**Figure 3:**
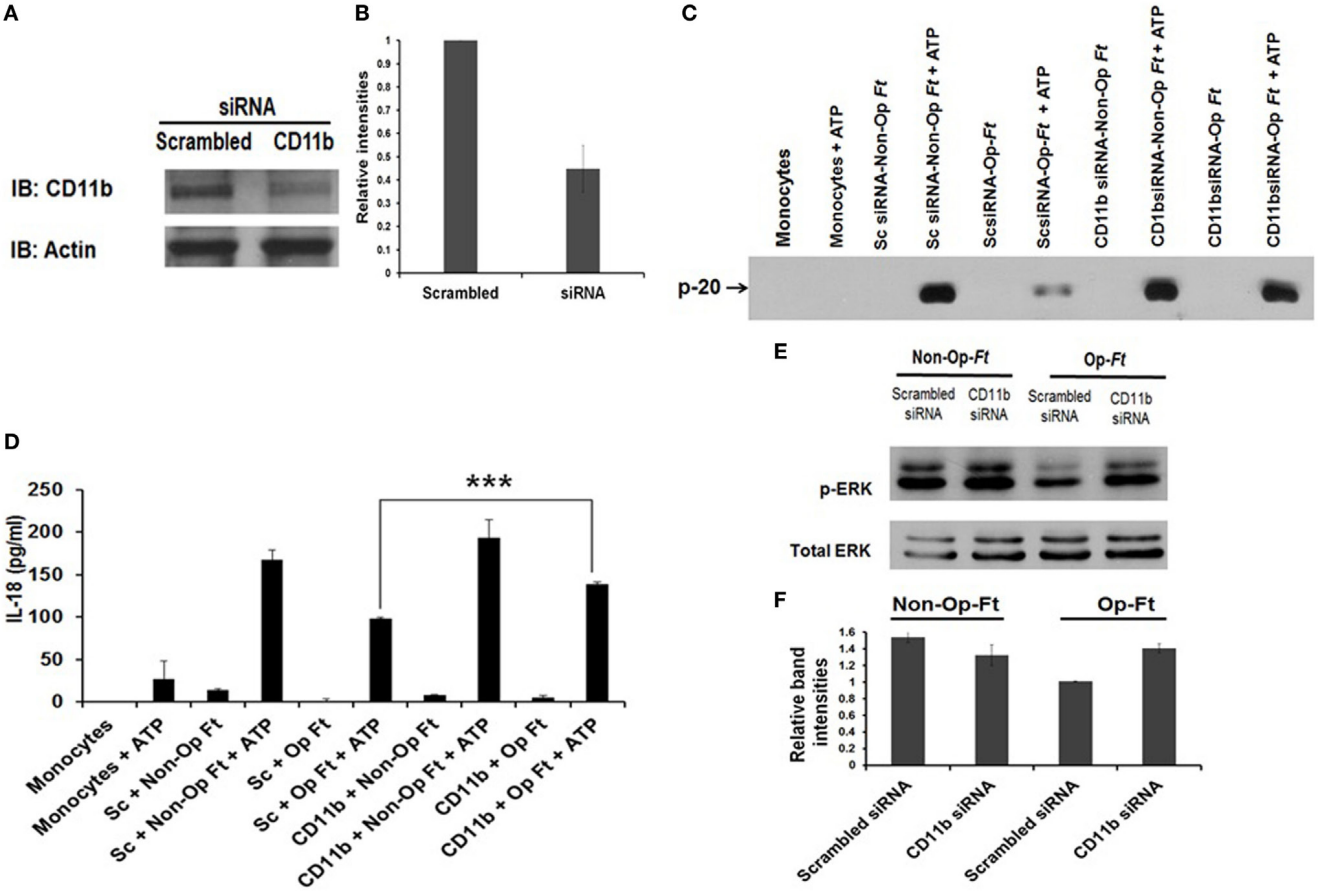
Inhibition of inflammasome priming by serum-pre-opsonized Schu S4 is mediated by complement receptor 3 (CR3). Monocytes were transfected with scrambled small interfering RNA (siRNA) or siRNA targeting CD11b, and 48 h later, transfected monocytes were infected with non-opsonized or serum pre-opsonized *Francisella* at an MOI of 100. Infection was synchronized by centrifugation at 250 × *g* for 10 min at 4°C for 5 min and then incubation at 37°C. After 30 min, ATP was added (5 mM) for an additional 30 min. CD11b knockdown effectiveness was determined by Western blot using CD11b specific Ab **(A)** and densitometry **(B)**. Supernatants were subjected to Western blot for caspase-1 cleavage **(C)**, IL-18 ELISA **(D)**, or to ERK activation **(E,F)** [relative ratio of pERK to total ERK from **(E)**]. Data are representative of three independent experiments performed in duplicate (mean ± SD) (***p < 0.001).

Although *Francisella* interacts with different host receptors during infection, dependent on the bacterial strain and mammalian species of macrophage used (5, 64), CR3 is a major phagocytic receptor, especially for primary human cells (54, 65). In addition, TLR2 plays a major role in generating pro-inflammatory responses during infection, mediated by the activation of MAPK and NF-κB signaling (14, 66–70). In this regard, inflammasome activation by *Francisella* has been associated with TLR2 on the host cells (14, 40) as well as ERK-mediated signaling (16, 40). C3 pre-opsonized Schu S4 uses CR3 to gain access into macrophages while at the same time inhibits TLR2-mediated host immune responses, as demonstrated by a reduction in activated ERK activation and pro-inflammatory cytokine production (54). Since C3 opsonization of Schu S4 significantly reduces inflammasome priming, we next sought to determine the role of CR3 in this process. We transfected human monocytes with scrambled siRNA (control) or siRNA targeting the CD11b component of CR3. CD11b knockdown effectiveness was determined by Western blot with approximately 65% reduction (Figures 3A,B). First, we confirmed our previous results with hMDMs (54) that Schu S4 attachment and uptake are markedly reduced in CD11b knockdown human monocytes compared to control siRNA (data not shown). Next, we infected CD11b siRNA transfected monocytes with serum-opsonized or non-opsonized Schu S4 and determined the levels of caspase-1 activation and IL-18 release. As expected, caspase-1 activation was decreased for serum-opsonized bacteria compared to non-opsonized bacteria and the scrambled siRNA control condition (Figure 3C). CD11b knockdown increased caspase-1 activation compared to scrambled siRNA control during serum-opsonized Schu S4 infection (Figure 3C). Consistent with this finding, IL-18 release was significantly increased in CD11b knockdown cells compared to scramble siRNA transfected cells (Figure 3D). Since MAPK/ERK activation is required for inflammasome priming, we examined the level of ERK activation under the above conditions. As expected, CD11b knockdown leads to a consistent increase in ERK activation during infection of serum-opsonized Schu S4 (Figures 3E,F). Taken together these data provide strong evidence that CR3, through its engagement of C3-opsonized bacteria, plays a critical role in inhibition of inflammasome priming by human monocytes.

### Uptake of Serum-Opsonized *Francisella* by Monocytes and hMDMs Leads to Increased RasGAP Activity

**Figure 4:**
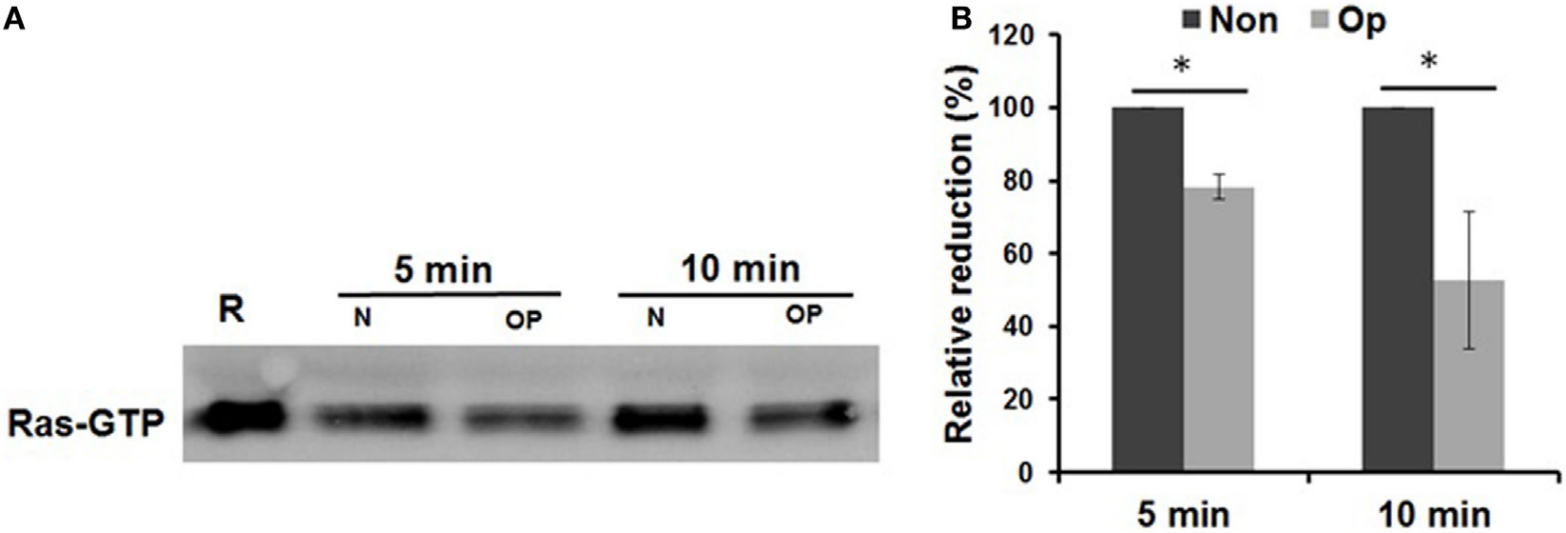
Serum opsonization of Schu S4 enhances Ras GTPase-activating protein (RasGAP) activity by human monocyte and monocyte-derived macrophages. Monocyte-derived macrophages monolayers were infected with non-opsonized or pre-opsonized bacteria at an MOI of 100. Non-infected control (R) was included. Infection was synchronized by centrifugation at 250 × *g* for 10 min at 4°C followed by incubation at 37°C. At the indicated time points, cell lysates were collected and subjected to a RasGAP pull down assay and Western blot **(A)**. Densitometry from three experiments is presented as percentage reduction of opsonized bacteria compared to non-opsonized bacteria **(B)**. Data are representative of three independent experiments. *p*-Values were calculated using one-way ANOVA for multiple comparisons and adjusted with Bonferroni’s correction (*p < 0.05).

We next sought to understand the mechanism(s) underlying inhibition of inflammasome priming by CR3 engagement of serum-opsonized Schu S4. The GAPs are implicated in downregulating the Ras superfamily of GTP-binding proteins, which activate MAPK signaling molecules (71). Thus, we examined the level of RasGAP activity in our infection model by measuring the level of GTP-bound Ras by immunoprecipitation (IP) followed by Western blot. Given the amount of protein necessary for the IP, we used hMDMs for this experiment. Our results (Figures 4A,B) show that the level of GTP-bound Ras is significantly reduced during infection with serum-opsonized Schu S4. These data indicate that uptake of serum pre-opsonized Schu S4 leads to increased RasGAP activity.

### Inhibition of Inflammasome Priming by Serum Pre-Opsonized Schu S4 Is Dependent on RasGAP

**Figure 5:**
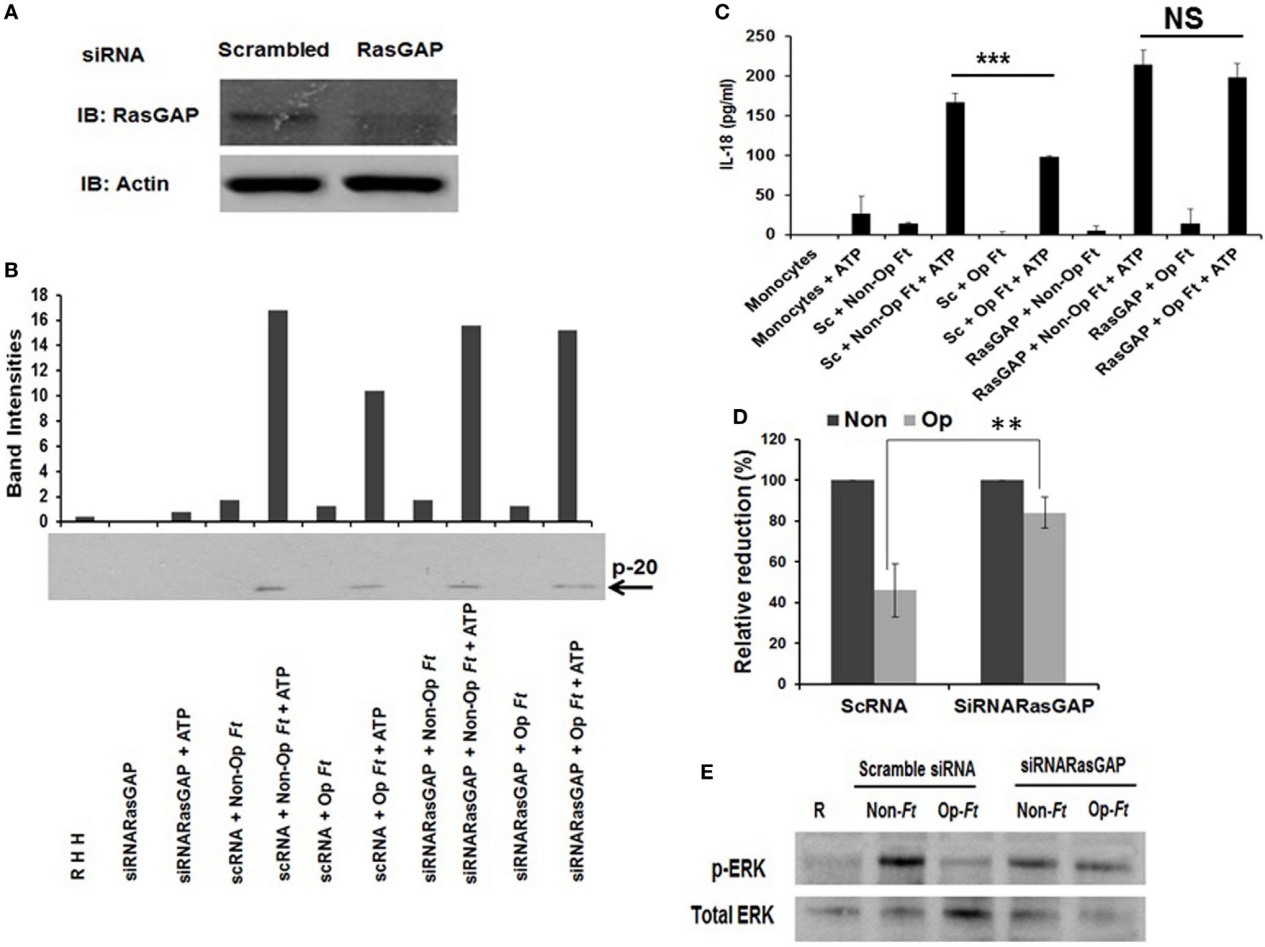
Ras GTPase-activating protein (RasGAP) regulates Sch S4-mediated inhibition of inflammasome priming. Human monocytes were transfected with scrambled small interfering RNA (siRNA) or siRNAs targeting RasGAP. RasGAP knockdown effectiveness was examined by Western blot using RasGAP Ab **(A)**. After 48 h transfected, monocytes were infected with non-opsonized or serum pre-opsonized bacteria (MOI of 100). Infection was synchronized at 4°C followed by incubation at 37°C for 30 min. ATP was added (5 mM) for an additional 30 min. Supernatants were collected and subjected to caspase-1 Western blotting **(B)** and IL-18 ELISA **(C)**. Cumulative data are shown for percentage IL-18 reduction, comparing pre-opsonized bacteria to non-opsonized bacteria **(D)**. Cell lysates were subjected to Western blotting using anti-phospho-ERK Ab **(E)**. Data are representative of three independent experiments performed in duplicate (mean ± SD). **p < 0.01; ***p < 0.001; NS, not significant.

To determine the role of RasGAP in limiting inflammasome priming mediated by serum-opsonized Schu S4, we transfected human monocytes with scrambled siRNA (control) or siRNA targeting RasGAP and subsequently infected cells with opsonized or non-opsonized Schu S4. RasGAP was effectively knocked down in human monocytes with ~80% reduction in RasGAP protein (Figure 5A). In control scrambled siRNA-treated cells, caspase-1 cleavage (Figure 5B; Figure S4 in Supplementary Material) and IL-18 release (Figures 5C,D) were increased using non-opsonized bacteria compared to opsonized bacteria as predicted. Most significantly, the inhibitory effect on inflammasome priming by serum-opsonized bacteria was reversed by RasGAP knockdown. Consistent with these data, ERK activation was increased by RasGAP inhibition during infection of serum-opsonized Schu S4 (Figure 5E). These data provide a causal link between increased RasGAP activity and inhibition of inflammasome priming following uptake of serum-opsonized Schu S4.

### Serum-Opsonized Schu S4 Activates Dok-1 Which Binds to RasGAP

**Figure 6:**
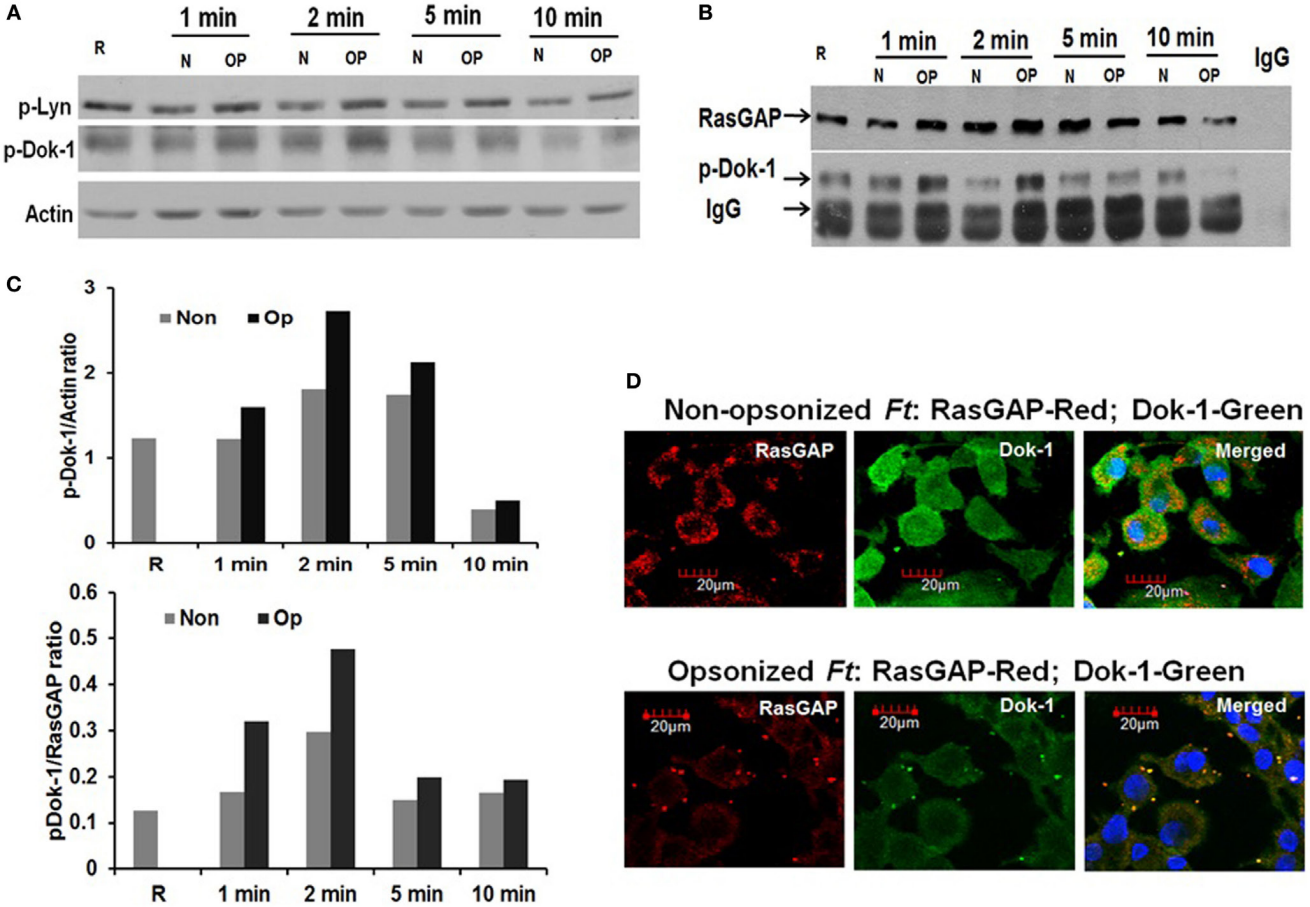
Dok-1 is phosphorylated during infection with serum-opsonized Schu S4 and co-localizes with Ras GTPase-activating protein (RasGAP). Monocyte-derived macrophage monolayers were infected with non-opsonized or pre-opsonized bacteria (MOI of 100). Infection was synchronized at 4°C followed by incubation at 37°C. At the indicated time points, cell lysates were collected and subjected to Western blot for phosphorylated Lyn and Dok-1 **(A)**. Phosphorylated Dok-1/actin band intensity ratio at different time points from **(A)** is shown in **(D)**. Association of RasGAP with phosphorylated Dok-1 was examined by immunoprecipitation using anti-RasGAP Ab **(B)**. Phosphorylated Dok-1/RasGAP band intensity ratio at different time points from **(B)** is shown in **(D)**. Data from **(A)** and **(B)** are representative of 3 independent experiments. Co-localization of RasGAP with Dok-1 was examined at the 5 min time point by confocal microscopy **(C)**. Data are representative photomicrographs from three independent experiments.

Adaptor protein Dok-1 belongs to the Dok protein family (Dok-1–Dok-7), which shares structural similarities characterized by a NH_2_-terminal pleckstrin homology (PH) domain and phosphotyrosine-binding (PTB) domain as well as an SH_2_ target motif in the COOH-terminal moiety’s tyrosine. Phosphorylation of Dok-1 by Src family kinases contributes to its inhibitory effects on the Ras-ERK signaling cascade directly or indirectly through RasGAP (72, 73). In this regard, we have previously shown that serum opsonization of Schu S4 increases Lyn kinase phosphorylation in human macrophages (54). We therefore simultaneously examined the phosphorylation of Lyn and Dok-1 in hMDMs. Consistent with our previous findings, Lyn phosphorylation was increased during infection of serum-opsonized bacteria compared to non-opsonized bacteria over a 10 min time course of study (Figures 6A,D). Increased Dok-1 phosphorylation was observed consistently between 1 and 5 min following infection with pre-opsonized bacteria, depending on the donor. Since phosphorylated Dok-1 serves as a docking site for RasGAP to potentiate GAP activity, we next determined whether activated Dok-1 binds to RasGAP using IP followed by Western blot. hMDMs were used to obtain sufficient protein for the IP. The results shown in Figures 6B,D indicate that activated Dok-1 binds to RasGAP during infection of opsonized Schu S4, particularly apparent at 1 and 2 min, suggesting that Dok-1’s ability to inhibit Raf/MEK-mediated ERK activation is at least in part *via* its binding to RasGAP. Consistent with the IP experiment, RasGAP co-localization with Dok-1 was increased during infection of serum pre-opsonized Schu S4 (Figure 6C). These results provide evidence for involvement of Dok-1 in the CR3-Ras-ERK signaling cascade mediated by serum pre-opsonized Schu S4.

## Discussion

The high virulence of *F. tularensis* in pneumonic tularemia is at least in part due to the ability of the bacterium to subvert and suppress host immune responses (6, 74–77). Virulent Schu S4 infection induces limited production of pro-inflammatory cytokines and antagonizes subsequent stimulation by TLR agonists (6, 77–81). CR3 is a major phagocytic receptor for this bacterium, particularly on human monocytes and macrophages (54, 65) and provides a safe entry for many intracellular bacterial pathogens including *F. tularensis* (41, 43, 54, 82–84). Our previous study showed that ligation of CR3 with serum pre-opsonized *Francisella* results in dampening TLR2-mediated ERK activation (54). While inhibition of ERK1/2 occurred as early as 5 min postinfection, the inhibition of p38 and the level of MKP-1 were not maximal until 30 min (54). The question remained as to what is the signaling cascade(s) immediately downstream of CR3 that leads to early inhibition of ERK1/2.

In response to bacterial infection, Ras can be activated through direct association with TLR2 (85), the primary receptor by which *Francisella* activates MAPK and NF-κB that leads to inflammasome priming and production of pro-inflammatory cytokines (14, 40, 66, 67, 70). Ras is a GTP-binding protein of the Ras superfamily involved in a wide range of important cellular processes *via* participation in the receptor tyrosine kinase (RTK)/Ras GTPase/MAPK signal transduction pathway (86, 87). Ras cycles between an inactive GDP-bound form and an active GTP-bound form. GTPase activity of Ras is tightly regulated. In resting cells, GTPase is maintained in its inactive GDP-bound state by its slow intrinsic rate of guanosine nucleotide dissociation. Upon stimulation, GTPase is either activated by GEFs *via* accelerating the dissociation of GDP, or deactivated by GAPs that catalyze the reaction (71). Our previous study showed that CR3-TLR2 crosstalk mediates immune suppression by limiting ERK activation in response to serum pre-opsonized Schu S4 (54) and that activation of inflammasome priming is dependent on ERK signaling (16). A subsequent mathematical model of CR3/TLR2 crosstalk in the context of *F. tularensis* infection predicted the involvement of RasGAP-mediated immune suppression by serum-opsonized Schu S4 (55).

Herein, we experimentally tested this model and provide evidence for a pathway that links CR3-mediated phagocytosis for *Ft* Schu S4 with immune suppression that involves early activation of RasGAP. We determined that ligation of CR3 on the phagocytic cells by pre-opsonized Schu S4 recruits RasGAP to the phagocytic site and increases RasGAP activity, which leads to inhibition of the Ras-ERK signaling cascade and suppression of inflammasome activation, as evidenced by increased inhibition of ERK1/2, limited caspase-1 cleavage, and IL-18 release. Importantly, to isolate inflammasome priming events (signal 1 in inflammasome activation) from the NLR recognition phase (signal 2) we used a 30 min priming window. At this time point, no IL-1β is synthesized, cleaved, or released. However, pro-IL-18 is constitutively expressed in monocytes and MDMs, allowing it to serve as an ideal substrate read out for early inflammasome activation events. We have therefore intentionally excluded IL-1β from our analyses. We propose that this focus on IL-18 thus allows for a careful dissection of this early signal 1 inflammasome activation time point.

Following phagocytosis, *Francisella* resides in a *Francisella*-containing phagosome and subsequently escapes into the cytosol where it rapidly multiplies and induces inflammasome activation (step 2) and pyroptosis as evidenced by mature caspase-1, IL-1β, and IL-18 release (5, 14, 26, 29–31). Phagosomal escape, TLR2-dependent expression of pro-inflammatory cytokines (e.g., pro-IL-1β), and bacterial DNA release are required for inflammasome activation mediated by host AIM2 (14, 32, 33), pyrin (34, 36), and NLRP3 (35, 38). Macrophages and dendritic cells infected with virulent Schu S4 secrete limited levels of the inflammasome-dependent cytokines IL-18 and IL-1β accompanied by weak caspase-1 activation compared to avirulent strains LVS and *Fn* (38, 88).

Inflammasome priming can be triggered by TLR agonists within minutes of stimulation (16, 17, 22, 23) and is dependent on ERK signaling and proteasome function (16). Our recent study showed that *Francisella* can prime inflammasome activation in human monocytes which express a constitutive level of pro-IL-18, an event dependent on TLR2-ERK signaling (40). In that study, we concluded that ERK activation and inflammasome priming are similar for virulent and avirulent species of *Francisella* and independent of MOI (40). However, it is important to highlight the difference to current study. The prior priming studies were conducted under different culture conditions (e.g., use of HI bovine serum) and did not study opsonization status. Our previous opsonization studies (38, 54, 89) and others (6, 27) have demonstrated that infection with virulent strain Schu S4 induces less TLR2-dependent immune responses than that of avirulent strain *Fn*, including activated ERK (54) and that ligation of CR3 with serum pre-opsonized *Francisella* resulted in dampening TLR2-mediated ERK activation in human cells (54). By contrast, complement-opsonized Schu S4 induced robust ERK activation in mouse macrophages (54) and CR3 has a protective role in systemic *F. tularensis* LVS infection in mice (90).

TLR2-induced ERK activation occurs through a previously characterized MyD88-independent pathway in which GTPase proteins Rac and Ras associate with the cytoplasmic domain of TLR2 and undergo rapid activation in response to bacterial stimuli (85, 91). The Raf-MEK-ERK pathway is a key downstream effector of Ras. It is well documented that RasGAP is a potent negative regulator of the Ras-MAPK signaling pathway (92, 93) and that membrane immobilization of Ras and RasGAP is important for this negative regulation (94–96). The molecular structure of RasGAP includes an amino-terminal region containing a Src homology 3 (SH3) domain flanked by two SH2 domains that associate with several phosphorylated tyrosine kinase receptors, the non-RTK v-Src, and Dok adaptor family proteins. There is a PH domain that binds phosphatidylinositol lipids (PIP2 and PIP3) within biological membranes, a calcium-dependent phospholipid-binding domain (C2), and the catalytic GAP domain located in the C-terminal region (93, 97, 98). We found that RasGAP activity increases upon infection by pre-opsonized Schu S4 compared to non-opsonized bacteria evidenced by a reduction in Ras-GTP level (Figures 4A,B). This increase in RasGAP activity is responsible for limiting the activation of ERK and inflammasome priming since knockdown RasGAP by siRNA abolished the effects (Figure 5). These data provide evidence that pre-opsonized *Ft* Schu S4 suppresses immune responses through CR3 at an early proximal step that involves recruitment of RasGAP to the membrane.

The activity of RasGAP requires adaptor proteins Dok-1 (p62) and Dok-2 for the negative regulation of Ras-ERK signaling (99), and TNF and nitric oxide production upon LPS treatment of macrophages, a step that is independent of TLR2 (99, 100). The Dok protein family has seven members (Dok-1–Dok-7) which share structural similarities characterized by N-terminal PH and PTB domains followed by SH2 target motifs in the C-terminal that serve as binding sites for the SH2 domains of RasGAP (99). Phosphorylation of tyrosine 295 and 361 of Dok-1 provides docking sites for the SH2-domain of RasGAP; however, phosphorylation of tyrosines 336 and 340 mediated by the non-RTK Src family members, Lyn or Fyn kinase, is essential for the negative regulation of Ras-ERK signaling (73, 101). In addition, Lyn/PI3K negatively regulates TLR2 and TLR4 signaling (102). Consistent with our previous publication (54), activation of Lyn kinase increased as early as 1 min upon ligation of CR3 by pre-opsonized Schu S4 (Figure 6A) as did Tyr361 phosphorylation of Dok-1 (Figure 6A), a docking site for RasGAP (73). Association of RasGAP with Dok-1 was increased upon infection by pre-opsonized Schu S4 compared to that of non-opsonized bacteria (Figure 6C). These data provide evidence for the involvement of Dok-1 in the CR3-RasGAP-MAPK signaling pathway in response to Schu S4 infection.

Complement receptor 3 is a major receptor for intracellular bacterial pathogens (45, 103–105) and immunosuppressive signaling events associated with CR3 have been examined in several studies (84, 106, 107). For example, ligation of CR3 by the surface virulent factor BAD1 of *Blastomycetes dermatitidis* results in TNF suppression and immune invasion (84), and infection of murine macrophages with *Mycobacterium avium* in C3-depleted medium resulted in a significantly higher level of TNF production (108). Therefore, it will be interesting to investigate whether these pathogens also employ similar signaling mechanisms related to CR3 in order to evade the immune system to enhance their survival during infection.

In summary, our studies have identified a new signaling mechanism that links CR3 with inhibition of inflammasome priming during serum-opsonized Schu S4 infection of human monocytes and macrophages. This involves early recruitment and activation of RasGAP, which reduces ERK activation and downstream signaling. CR3-mediated immune suppression is an important strategy used by *F. tularensis* for its pathogenicity in humans. These results have broader implications for the pathogenesis of other intracellular pathogens that utilize CR3 for phagocytosis.

## Ethics Statement

This study was carried out in accordance with the recommendations of The Ohio State University Institutional Review Board with written informed consent from all subjects. All subjects gave written informed consent in accordance with the Declaration of Helsinki. The protocol was approved by The Ohio State University Institutional Review Board.

## Author Contributions

Conceived and designed experiments, analyzed the data, and wrote the paper: LS, KH, MR, MW, and MG. Performed the experiments: KH, HC, and MR.

## Conflict of Interest Statement

The authors declare that the research was conducted in the absence of any commercial or financial relationships that could be construed as a potential conflict of interest.

## Supplementary Material

Figure S1 Serum mediates immune suppression of human monocytes by *Francisella*. Approximately 10^6^ monocytes were infected with either non-opsonized (N) or autologous serum pre-opsonized (Op) *F. tularensis* (*Ft*) or *F. novicida* (*Fn*) (MOI = 100). At the indicated time points, cells were collected, lysed, and subjected to Western blot using phosphor ERK and total ERK Antibodies. Data are representative of three independent experiments.

Figure S2 Full blot of caspase-1 for Figure 1A.

Figure S3 Full blot of caspase-1 for Figure 2A.

Figure S4 Full blot of caspase-1 for Figure 5B.
