# Supplementary figures and images for: Complement Receptor 3-Mediated Inhibition of Inflammasome Priming by Ras GTPase-Activating Protein During Francisella tularensis Phagocytosis by Human Mononuclear Phagocytes
Source: Front Immunol. 2018 Mar 26;9:561. doi: 10.3389/fimmu.2018.00561 (PMC5879101; doi:10.3389/fimmu.2018.00561)

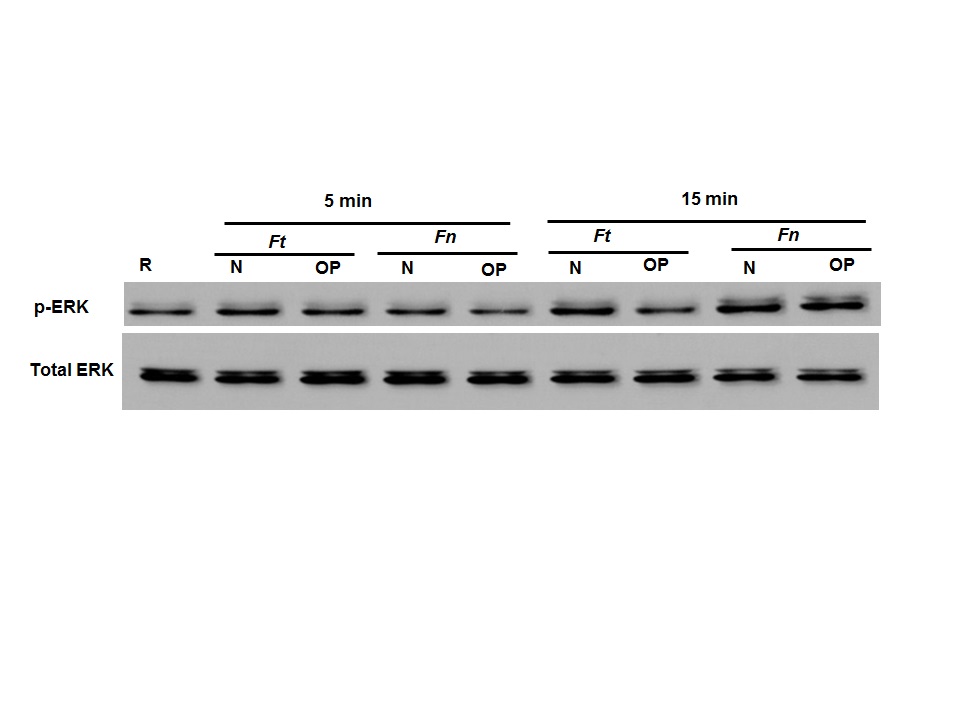

Supplement: Figure S1 — Serum mediates immune suppression of human monocytes by Francisella. Approximately 106 monocytes were infected with either non-opsonized (N) or autologous serum pre-opsonized (Op) F. tularensis (Ft) or F. novicida (Fn) (MOI = 100). At the indicated time points, cells were collected, lysed, and subjected to Western blot using phosphor ERK and total ERK Antibodies. Data are representative of three independent experiments. [file image_1.jpg]

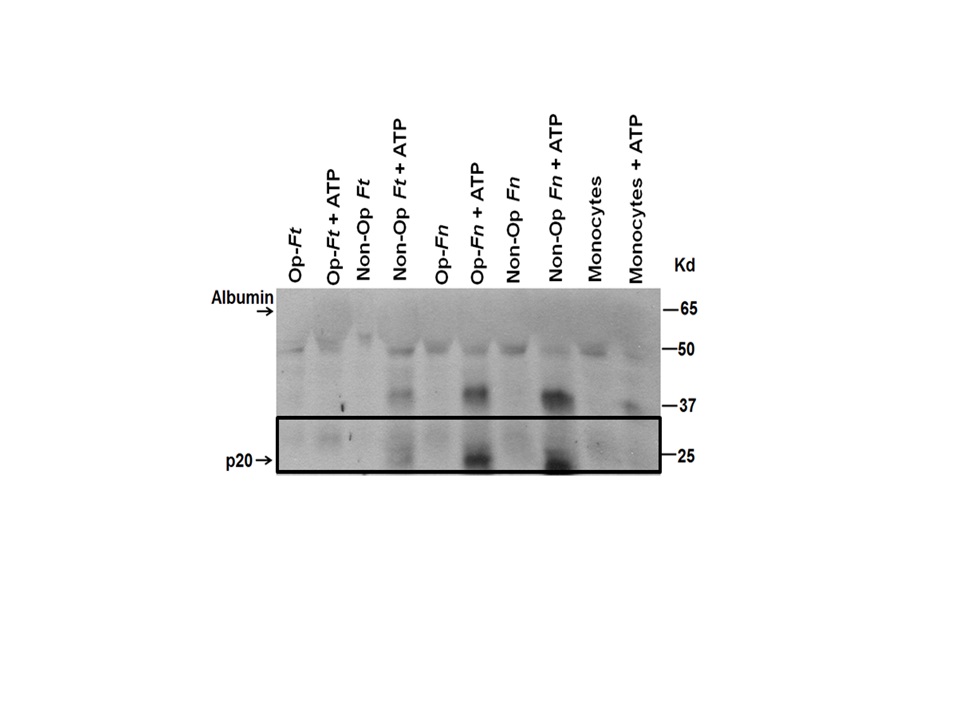

Supplement: Figure S2 — Full blot of caspase-1 for Figure 1A. [file image_2.jpg]

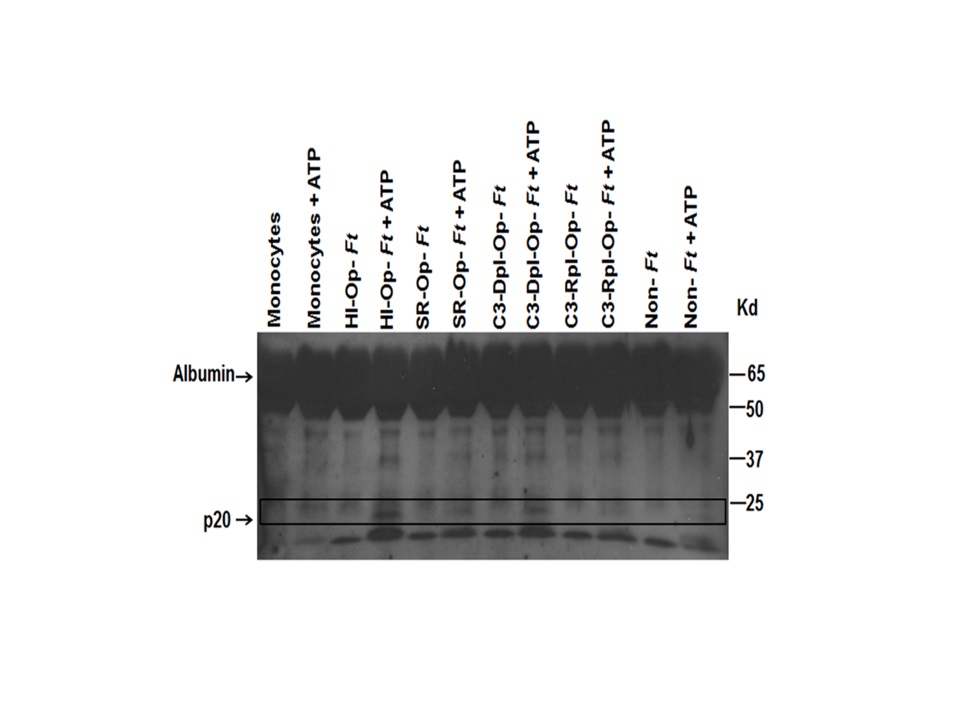

Supplement: Figure S3 — Full blot of caspase-1 for Figure 2A. [file image_3.jpg]

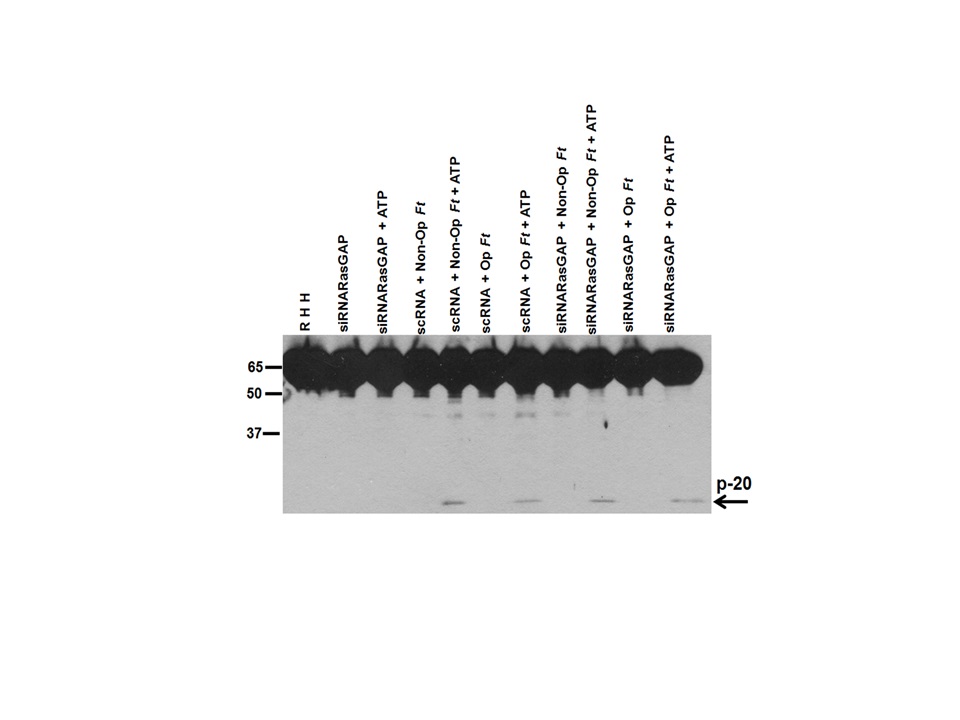

Supplement: Figure S4 — Full blot of caspase-1 for Figure 5B. [file image_4.jpg]
